# Imaging in juvenile idiopathic arthritis — international initiatives and ongoing work

**DOI:** 10.1007/s00247-017-4054-z

**Published:** 2018-01-13

**Authors:** Charlotte M. Nusman, Laura Tanturri de Horatio, Robert Hemke, E. Charlotte van Gulik, Lil-Sofie Ording Müller, Clara Malattia, Derk Avenarius, Paolo Toma, Johannes Roth, Nikolay Tzaribachev, Silvia Magni-Manzoni, Mario Maas, Andrea S. Doria, Karen Rosendahl

**Affiliations:** 1 Department of Pediatric Hematology, Rheumatology, Immunology and Infectious Disease, Emma Children’s Hospital, Academic Medical Center, 1100 Amsterdam, the Netherlands; 2 Department of Radiology, Academic Medical Center, 1100 Amsterdam, the Netherlands; 3 Department of Imaging, Bambino Gesù Children’s Hospital, Rome, Italy; 4 Department of Radiology and Intervention Unit for Paediatric Radiology, Oslo University Hospital, Ullevål, Oslo, Norway; 5 Pediatria 2- Reumatologia Istituto Giannina Gaslini, Genova and Department of Pediatrics, University of Genova, Genova, Italy; 6 Department of Radiology, University Hospital of North Norway, Tromsø, Norway; 7 Division of Pediatric Rheumatology, Children’s Hospital of Eastern Ontario, University of Ottawa, Ottawa, Canada; 8 Pediatric Rheumatology Research Institute (PRI), Bad Bramstedt, Germany; 9 Pediatric Rheumatology, Bambino Gesù Children’s Hospital, Rome, Italy; 10 Department of Radiology, SickKids Hospital, Toronto, Canada; 11 Department of Radiology, Haukeland University Hospital, Bergen, Norway; 12 Department of Clinical Medicine, K1, University of Bergen, Bergen, Norway

**Keywords:** Children, Juvenile idiopathic arthritis, Magnetic resonance imaging, Radiography, Ultrasound

## Abstract

Imaging is increasingly being integrated into clinical practice to improve diagnosis, disease control and outcome in children with juvenile idiopathic arthritis. Over the last decades several international groups have been launched to standardize and validate different imaging techniques. To enhance transparency and facilitate collaboration, we present an overview of ongoing initiatives.

## Introduction

Juvenile idiopathic arthritis is the most common chronic rheumatic disease in childhood. The diagnosis is based on clinical features, including onset before 16 years of age, persistence of arthritis in the same joint(s) for at least 6 weeks and the exclusion of other causes of arthritis.

**Fig. 1:**
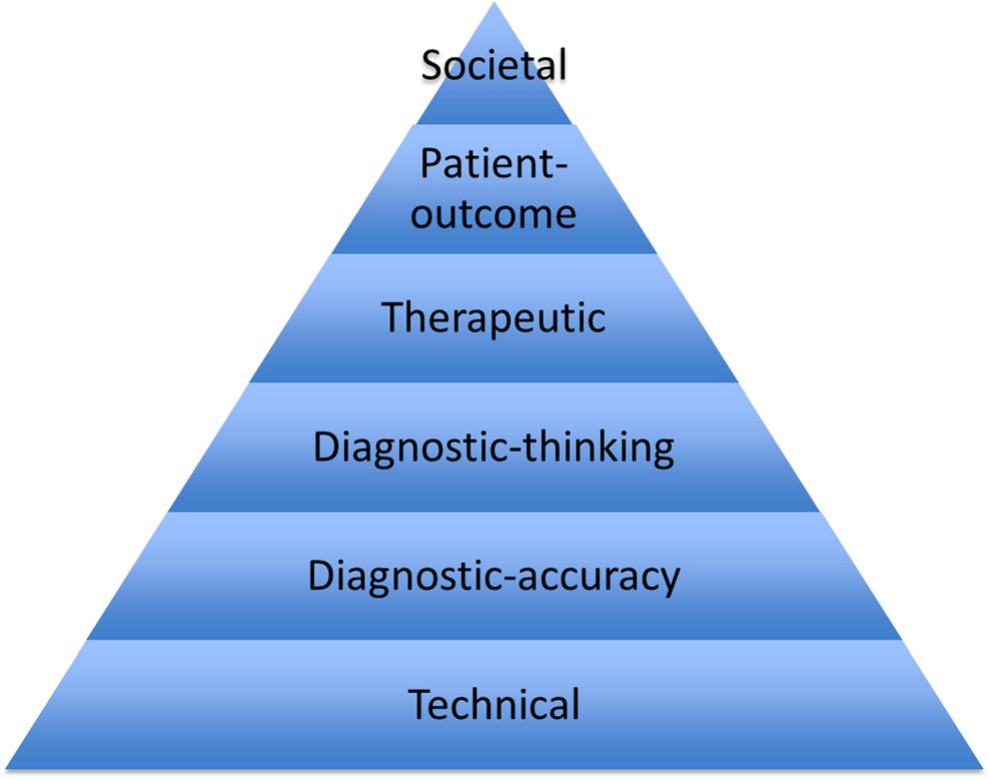
Levels of efficacy of diagnostic imaging reflected in the pyramid of Thornbury [1]

In recent years, imaging has become increasingly important to confirm diagnosis, monitor disease activity and predict the disease course and outcome in children with juvenile idiopathic arthritis. Several criteria or filters, such as those outlined in the Thornbury pyramid and in the OMERACT (Outcome Measures in Rheumatology Clinical Trials) filters, are being used to determine whether an imaging technique holds sufficient quality for accurate interpretation. The efficacy criteria of Thornbury [1] provide six levels of efficacy where every level presupposes the preceding ones (Fig. 1), while, according to OMERACT filters 1.0 and 2.0, an outcome measure should be feasible, correct (“truth”) and have the ability to discriminate among different grades of disease [2]. In the following series of papers we refer to Thornbury’s quality criteria when describing each method and joint, i.e. technical feasibility (is image quality sufficient?), diagnostic accuracy (is the variability of the findings within acceptable limits for clinical and research purposes?), diagnostic thinking (clinical validity; do imaging findings truly reflect disease activity or extent?), therapeutic and patient outcome (do findings guide disease monitoring and improve outcome?) and societal efficacy such as cost-effectiveness analysis.

During the last decades great efforts have been made to improve the quality of diagnostic imaging and to reach consensus on which method and scoring systems to use. This is particularly important when performing and comparing the results of clinical trials. We give an overview of ongoing international initiatives addressing imaging in juvenile idiopathic arthritis, their main focus and achievements (Table 1, references [3–29]). While some of the groups focus on all available imaging modalities, others specialize on magnetic resonance imaging (MRI) or ultrasound. Similarly, some perform research while others take on a more advisory role, although there is substantial collaboration across groups (Table 1).

**Table 1:** Ongoing international initiatives addressing imaging in juvenile idiopathic arthritis

Group	Principal investigator(s)	Since (year)	Joints	Goal (review or original research)	References	Rheumatologist/radiologist
Multiple modalities						
ACR Pediatric Rheumatology Imaging Working Group	Nikolay Tzaribachev Johannes Roth	2012	All	Informative platform	None	(Pediatric) rheumatologists
EULAR-PRES	Clara Malattia Philip Conaghan	2015	All	Review	[3]	Both
ESSR arthritis subcommittee	Iwona Sudol-Szopinska Lennart Jans	2003	All	Review	[4, 5]	Both
Health-e-Child	Karen Rosendahl	2006	Hip, wrist, whole body	Original research	[6–15]	Both
PRES imaging working party	Silvia Magni-Manzoni	2011	All	Both	None	Both
MD Paedigree	Clara Malattia Laura Tanturri de Horatio Silvia Magni Manzoni	2013	Ankle	Original research	[16]	Both
ESPR MSK task force	Karen Rosendahl	2007	All	Recommendation	None	(Pediatric) radiologists
Norwegian JIA Study	Karen Rosendahl	2011	TMJ	Both	[17]	Both
MRI only						
OMERACT MRI in JIA special interest group — large joints	Andrea Doria Robert Hemke	2011	Knee	Both	[18–21]	Both
OMERACT MRI in JIA special interest group — small joints	Andrea Doria Mario Maas Charlotte Nusman	2011	Wrist	Both	[15, 18–20, 22]	Both
OMERACT MRI in JIA special interest group — TMJ	Andrea Doria Marion van Rossum Mirkamal Tolend	2011	TMJ	Both	[18, 23]	Both
ANOME	Karen Rosendahl Mario Maas	2013	Wrist, knee, hip	Both	[15, 19, 22]	Both
Ultrasound only						
OMERACT US task force — pediatric group	Paz Collado	2010	All	Both	[24–28]	(Pediatric) rheumatologists
PANLAR ultrasound group	Marwin Gutierrez Johannes Roth	2016	All	Both	[29]	(Pediatric) rheumatologists
CARRA ultrasound group	Johannes Roth Ed Oberle	2015	All	Both	None	(Pediatric) rheumatologists

*ACR* American College of Rheumatology, *ANOME* Amsterdam November Meeting, *CARRA* Childhood Arthritis and Rheumatology Research Alliance, *ESPR* European Society of Paediatric Radiology, *ESSR* European Society of Musculoskeletal Radiology, *EULAR* European League Against Rheumatism, *JIA* juvenile idiopathic arthritis, *MRI* magnetic resonance imaging, *MSK* musculoskeletal, *OMERACT* Outcome Measures in Rheumatoid Arthritis Clinical Trials, *PANLAR* Pan-American League of Rheumatology Associations, *PRES* Pediatric Rheumatology European Society, *TMJ* temporomandibular joint, *US* ultrasound

## Multi-modality groups

### American College of Rheumatology Pediatric Rheumatology Imaging Study Group

The American College of Rheumatology (ACR) Pediatric Rheumatology Imaging Study Group was established in 2010 with the aim to promote imaging in pediatric rheumatology. The study group follows ACR guidelines and provides updates on current topics and advances in imaging research in pediatric rheumatology on a yearly basis to pediatric rheumatologists and allied health care professionals. Regularly, different specialists and researchers deliver new research data on a certain topic from different perspectives (e.g., MRI, ultrasound, computed tomography [CT]). The study group promotes research and wide participation of interested colleagues in current and future research projects. The study group also provides a link between other pediatric imaging working groups in rheumatology (e.g., OMERACT, Pediatric Rheumatology European Society). The future scope of the study group is to continue to link interested health care providers to imaging research in pediatric rheumatology and crosslink various working groups.

### European League Against Rheumatism-Pediatric Rheumatology European Society

A European League Against Rheumatism (EULAR)–Pediatric Rheumatology European Society (PRES) task force was convened in 2013 to establish recommendations on the use of imaging in the diagnosis and management of juvenile idiopathic arthritis in clinical practice. The task force comprises pediatric rheumatologists, rheumatologists experienced in imaging, radiologists, methodologists and patients from nine countries. Initially, the task force performed a detailed systematic search of the published literature on the use of imaging in juvenile idiopathic arthritis. Included studies were evaluated for risk of bias and applicability using the Quality Assessment of Diagnostic Accuracy Studies (QUADAS)-2 tool [30]. The task force included studies using conventional radiography, ultrasound, MRI, CT, scintigraphy and positron emission tomography (PET). Following presentation of the data from the literature review, the task force developed nine points to consider (supporting data were not sufficient to produce “recommendations”) for the role of imaging in juvenile idiopathic arthritis. The nine points are based on the best available evidence from the literature and on the opinion of the international panel of experts and encompass the role of imaging in making a diagnosis of juvenile idiopathic arthritis, detecting inflammation and damage, predicting outcome and response to treatment, and monitoring disease progression and remission [3]. The available evidence for each recommendation was scored by the experts according to the Oxford Centre for Evidence-based Medicine (CEBM) level of evidence. The task force also produced an agenda for future research in the field.

### European Society of Musculoskeletal Radiology (ESSR) Arthritis Subcommittee

In 2003 the European Society of Musculoskeletal Radiology (ESSR) launched its arthritis subcommittee. The committee promotes the standardization of the use of imaging in arthritis assessment. It proposes structured protocols for performing, interpreting and quantifying imaging findings. Recommendations are written concerning the use of MRI in musculoskeletal rheumatic diseases including juvenile idiopathic arthritis [4] and, more disease-specific, regarding imaging of axial (juvenile) spondylarthritis [5]. “Arthritis imaging” sessions are held annually at the ESSR meetings, which have been organized in collaboration with the EULAR since 2012. Since 2015 the subcommittee has taken part in organizing the annual Sports and Arthritis MSK Conference.

### Health-e-Child

The Health-e-Child radiology group was established in 2006, emerging from a large longitudinal multi-center project (Health-e-Child, EU-FR6) aimed at combining clinical, laboratory and radiologic data of a large number of children with juvenile idiopathic arthritis. The development of an MRI scoring system for wrist and hip involvement in juvenile idiopathic arthritis was among the principal objectives of this work, which involved the collaboration of four large pediatric centers: Great Ormond Street Hospital, London (GOS); Hopital Necker Enfants Malades, Paris (NEM); Ospedale Gaslini, Genoa; and Ospedale Bambino Gesu, Rome. During 2010, Norwegian centers joined the group, focusing on normative standards for wrist MRI. Standards for wrist ultrasound are underway. To date, the group has made contributions of MR-wrist scoring systems [6–9], normative standards for wrist MRI [10–14] and timing of intravenous contrast in wrist MRI examinations [15]. Currently, the group is undergoing novel work on radiographic and MRI scoring systems for hip involvement in juvenile idiopathic arthritis and normative standards for whole-body MRI. The group collaborates with Amsterdam November Meeting (ANOME), the OMERACT special interest group on MRI in JIA, the ESSR, the European Society of Paediatric Radiology and the Norwegian Juvenile Idiopathic Arthritis Study.

### Paediatric Rheumatology European Society Imaging Working Party

The Imaging Working Party was initiated in 2015 at the Paediatric Rheumatology European Society (PRES) annual meeting in Belgrade, as a response to the emerging need within the PRES membership to share experiences with and expectations of imaging. The aim of the PRES Imaging Working Party has been defined as the promotion of knowledge, research and education on imaging within the PRES frame. The group is open to all PRES members and people who share its aim and activities, summarized as “meet and work.” Initially 9 people from 5 European countries met, increasing to 22 colleagues from 13 countries at the PRES meeting in 2016. An update on the most recent advances in research and clinical applications of imaging in pediatric rheumatology is provided at every annual meeting. The group actively encourages international courses of imaging in children. Further, it promotes collaborative research projects with involvement of pediatric rheumatologists, pediatric radiologists, care-givers and scientists interested in imaging in children with rheumatologic diseases.

### MD Paedigree

MD Paedigree, a model-driven European pediatric digital repository, is a clinically led virtual physiological human multicenter project (2012–2016) funded by the European Union 7th Framework Programme for Research and Technological Development (EU FP7). MD Paedigree develops patient-specific computer-based predictive models of various pediatric diseases, including juvenile idiopathic arthritis, thus providing newly defined workflows for personalized predictive medicine at the point of care. The management of juvenile idiopathic arthritis has changed dramatically over the last two decades with the development of new therapeutic agents and the shift toward early aggressive interventions. Early identification and treatment of patients at higher risk to develop joint destruction and serious functional impairment has become a high priority. In the frame of this project clinical, immunological, metagenomic and imaging data have been collected in a prospective cohort of new-onset juvenile idiopathic arthritis patients. The potential role of an extensive ultrasound assessment (including wrists, metacarpophalangeal and interphalangeal joints, elbows, hips, knees and ankles) in predicting disease severity and response to treatments has been explored. Patients with clinical ankle involvement were also investigated by MRI, gait cycle analysis and whole-body MRI. MD Pedigree integrated the data from these evaluations to build a patient-specific finite element model of the joints to explore the role of the biomechanical determinants on the disease severity and structural damage progression [16]. The group is also developing and validating an MRI scoring system to assess disease activity and damage in the ankle in children with juvenile idiopathic arthritis.

### The Norwegian Juvenile Idiopathic Arthritis Study

The Norwegian Juvenile Idiopathic Arthritis Study uses a cross-disciplinary, longitudinal prospective design and includes pediatric radiologists, rheumatologists, pedodontists (i.e. a dentist specializing in the care of children’s teeth), maxillo-facial surgeons, and physicists from Norway, with input from colleagues in Denmark and Canada. It combines clinical, laboratory and radiologic data from a large number of children with juvenile idiopathic arthritis, to validate, refine and further explore different imaging techniques used for the assessment of temporomandibular joint involvement in children with juvenile idiopathic arthritis. The focus is on advanced MRI techniques and image analysis [17].

## Groups focusing on magnetic resonance imaging only

### OMERACT MRI in Juvenile Idiopathic Arthritis Special Interest Group

In 2011, prior to the Outcome Measures in Rheumatology (OMERACT) 11 meeting, a special interest group on MRI in juvenile idiopathic arthritis was formed. During the OMERACT 11 meeting, participants agreed that the special interest group would concentrate on the development and further refinement of MRI scoring systems. Because juvenile idiopathic arthritis includes different subtypes with a different clinical picture, it was decided that the special interest group would focus on the development of MRI as an outcome measure in juvenile idiopathic arthritis at three joint levels: (1) large joints (knees and ankles), (2) small joints (wrists and hands) and (3) temporomandibular joints [18]. Recently, a fourth group focusing on whole-body MRI was established. The members of the OMERACT special interest group on MRI in juvenile idiopathic arthritis consists of (pediatric) rheumatologists, pediatric/musculoskeletal radiologists, and dentists (temporomandibular joint subgroup).

The different subgroups are focusing on the establishment of standardized imaging protocols, definitions and MRI scoring systems. Also, reliability studies have been performed following face-to-face meetings. Especially the small joints subgroup has close collaboration with other international initiatives (ANOME, Health-e-Child).

Future steps of the special interest group on MRI in juvenile idiopathic arthritis will be: (1) to further refine and validate the different MRI scoring systems (including reference atlases), (2) to develop an MRI atlas of healthy joints at different ages and (3) to perform studies on the correlation between MRI and clinical characteristics of disease status in juvenile idiopathic arthritis (“truth”).

### Amsterdam November Meeting

The Amsterdam November Meeting (ANOME) was established in 2013 and is held annually. It includes pediatric rheumatologists, pediatric radiologists, musculoskeletal radiologists and research fellows/students from different initiatives mentioned in the current paper (OMERACT MRI in juvenile idiopathic arthritis special interest group, Health-e-Child). ANOME aims to “find the truth” in imaging juvenile idiopathic arthritis, through literature reviews and research projects. Currently, the main focus is wrist and knee imaging, using the principle “describe, not explain” [15, 19, 22]. Future focus will be to examine (construct) validation of MRI scores, hip imaging, and to enhance international collaboration among groups within the field.

### European Society of Paediatric Radiology Musculuskeletal Task Force

The group was established in 2004, aiming to promote evidence-based musculoskeletal imaging in children. The group plays an advisory role with respect to imaging strategies for juvenile idiopathic arthritis within the pediatric radiology society (www.espr.org).

## Groups specific to ultrasound

### OMERACT ultrasound Task Force–Pediatric Group

The pediatric group of the OMERACT US task force was established at the American College of Rheumatology meeting in Atlanta in 2010 to address musculoskeletal US definitions of normal and pathological findings specific for children. Currently, the group includes more than 30 people from several countries in Europe, North America and Central America. The overall aim is to investigate and test musculoskeletal US in pediatric rheumatology through the OMERACT filter [2, 31].

The group first conducted a systematic literature review on US in detecting synovitis in juvenile idiopathic arthritis and a survey on the use and expectations on pediatric musculoskeletal ultrasound [24, 25]. Next, the group provided US definitions of normal joint components in children [26] and proposed standardized image acquisition for four joints [28]. Additional definitions for joint components, not previously described, were further proposed. A wider group of pediatric musculoskeletal ultrasound experts tested the available definitions for applicability, intra- and interobserver agreement, and validation (manuscript in preparation). Then, the group identified US definitions for physiological vascularization and ossification grade in healthy children/adolescents (Windshall et al. — submitted for publication). Finally, the group defined and validated US elementary lesions in juvenile idiopathic arthritis and developed preliminary definitions for synovitis [27]. Ongoing steps are to develop and validate a semiquantitative scoring system (0–3) for synovitis in children, for both B-mode and Doppler US, and to test the reliability of the scoring system and its sensitivity to change.

### Pan-American League of Rheumatology Associations Ultrasound Group

Wthin the Pan-American League of Rheumatology Associations (PANLAR) Ultrasound Group, a pediatric subgroup, was formed several years ago. The goals of the pediatric subgroup are to foster the dissemination of ultrasonography in pediatric rheumatology practice through education and presentations at professional meetings. In 2016 the group started to contribute to international efforts of standardization in pediatric musculoskeletal ultrasonography through the dissemination, evaluation and further refinement of definitions, acquisition standards and scoring systems for the various structures relevant in pediatric rheumatologic disease.

### Childhood Arthritis and Rheumatology Research Alliance Ultrasound Group

The Childhood Arthritis and Rheumatology Research Alliance (CARRA) is a well-established group of pediatric rheumatologists in North America focusing on collaborative research to improve standards of care for patients with pediatric rheumatic diseases.

The increasing importance of musculoskeletal ultrasound as an outcome measure in research and for clinical care is reflected in the establishment of a musculoskeletal ultrasound group within CARRA in 2015. The goals of this group are to enhance ultrasound skills among CARRA members through training and the validation of ultrasonography as an outcome measure. The first validation project was started in 2016, is ongoing and is focusing on acquisition standards as well as scoring of the knee joint.

## Conclusion

The overview provided in this paper gives insight into the different ongoing international initiatives working on imaging in juvenile idiopathic arthritis. To date, each individual collaborative initiative has had important influence on improving our knowledge and thereby patient outcomes in juvenile idiopathic arthritis. The overview further enhances transparency and enables groups to combine their forces to reach the universal goal: improving the quality of imaging in children with juvenile idiopathic arthritis in order to improve their quality of life.
